# Bayesian inference of the viscoelastic properties of a Jeffrey’s fluid using optical tweezers

**DOI:** 10.1038/s41598-021-81094-x

**Published:** 2021-01-21

**Authors:** Shuvojit Paul, N Narinder, Ayan Banerjee, K Rajesh Nayak, Jakob Steindl, Clemens Bechinger

**Affiliations:** 1 Fachbereich Physik, Universität Konstanz, 78457 Konstanz, Germany; 2 Indian Institute of Science Education and Research Kolkata, Kolkata, India

**Keywords:** Materials science, Statistical physics, thermodynamics and nonlinear dynamics, Characterization and analytical techniques

## Abstract

Bayesian inference is a conscientious statistical method which is successfully used in many branches of physics and engineering. Compared to conventional approaches, it makes highly efficient use of information hidden in a measured quantity by predicting the distribution of future data points based on posterior information. Here we apply this method to determine the stress-relaxation time and the solvent and polymer contributions to the frequency dependent viscosity of a viscoelastic Jeffrey’s fluid by the analysis of the measured trajectory of an optically trapped Brownian particle. When comparing the results to those obtained from the auto-correlation function, mean-squared displacement or the power spectrum, we find Bayesian inference to be much more accurate and less affected by systematic errors.

## Introduction

Several fluctuation dominated physical phenomena, like, Brownian motion in various fluids, radioactive decay of sub-atomic particles, current fluctuations in resistors, etc^1,2^, are modeled by stochastic processes. To obtain the relevant parameters characterizing such processes, typically one analyzes a time series of a measured quantity. Examples are the measurement of resistance from the time series of current fluctuations or the inference of rheological parameters (viscosity, time constant) of a fluid from the trajectories of Brownian particles. When analyzing such data, often one only makes partial use of the information provided by the data, e.g., the correlation function, power spectral density in the context of Brownian motion, to an equation which is assumed to describe the underlying process, mainly using least-squares fitting routines. Often such technique delicately depends on the range of the data used for the comparison, and therefore the estimated results are rather unreliable. Bayesian inference with proper likelihood function added to the prior knowledge about the parameters can provide reliable and efficient measurements of the probable values of the parameters with the related uncertainties, for a given set of data. This method has been proved more detailed, advanced and reliable compared to least-squares fittings or the frequentist approaches^3–6^. Also, it makes an optimal use of the whole sample path and therefore does not require further guidance for the measurements^5,6^. Note that also model-free methods exist which allow to extract relevant microrheological parameters^7^. Often, however, specific models such as the Jeffrey’s model are used because they are based on simple and general mechanical concepts^8–15^.

Recently, Bayesian probability theory has been used for microrheological measurements, in viscous fluids and it has been shown that such methods are less affected by systematic noise present in the data^4,16^. However, to the best of our knowledge, so far, this theory has not been applied to viscoelastic fluids. Because most fluids and systems relevant in context of technical and biological entities are viscoelastic^17–20^, there exists a strong interest in the scientific community to efficiently measure the rheological properties of such fluids. In contrast to purely viscous fluids, viscoelastic fluids combine viscous and elastic properties which are often quantified by the imaginary and the real parts of the complex shear modulus $G^{*}(\omega )$ respectively^21–23^. In case of passive microrheology, $G^{*}(\omega )$ is calculated by Fourier transforming the mean-squared displacement (MSD) of a micro probe particle and using the Stokes-Einstein relation^22,23^. However, in case of limited data Fourier transformations are not trivial since interpolation and extrapolation from data points can easily yield far-reaching artifacts^22^.

Several models have been developed to describe viscoelastic fluids^21–26^, e.g., the Maxwell model , the Jeffrey’s model or the generalized Maxwell model^10,21,27,28^. Among these, Jeffrey’s model is extensively and successfully employed for understanding a wide class of experimental details^8–10,29–31^. Contrary to viscous fluids, the motion of a particle in a viscoelastic fluid exhibits memory effects, i.e., it is a non-Markovian process^23,24^. Nevertheless, Markovianity can be regained at the cost of additional degrees of freedom describing a multivariate Ornstein-Uhlenbeck process^32,33^. For such stationary, Gaussian and Markovian processes, the likelihood function which corresponds to discretely observed sample paths can be exactly calculated in terms of the process parameters. Using the Bayes’ theorem, the probable parameter values concealed in given discrete observations can be obtained with the corresponding probabilities^33,34^. Such a Bayesian theory based time domain method is thorough, accurate and avoids limitations such as non-trivial Fourier transformations, data range dependent uncertainty observed in the measurement from the auto-correlation function (ACF), power spectral density (PSD), mean-squared displacement function (MSD) etc.

Here, we demonstrate a Bayesian inference based technique to evaluate the rheological properties of a Jeffrey’s fluid from the partial observation of the position fluctuation of an optically trapped Brownian particle. We calculate the likelihood function related to the probe’s trajectory and estimate the most probable parameter values and their standard errors by numerically maximizing the likelihood with respect to the parameters. Further, we compare our method with the measurements from the position ACF and show that the estimations obtained from the proposed method are more reliable compared to the regular measurements from the ACF. Note that PSD and MSD are directly linked to the ACF, and hence for comparison, the analysis of only one among these is sufficient. We also show numerically and experimentally that both the methods lead to the same estimations for a given time series provided a least-squares fitting is conducted carefully over a certain data range. Furthermore, we also investigate how the length of the time series and the sampling time step affects the accuracy of the rheological parameters, similar to recent work^35^ where authors quantitatively determine the efficacy of their method.

Finally, we compare our results with macroscopic rheological measurements for various viscoelastic fluids and obtain good agreements.

## Theory

We describe the one dimensional Brownian motion of a microscopic particle of mass *m* confined in a harmonic potential of stiffness *k* in a homogeneous and isotropic viscoelastic medium by using the generalized Langevin equation (GLE)^24,32,34^:

1 $$\begin{aligned} m\ddot{x}(t)=-kx(t)-\int _{-\infty }^{t}\Gamma (t-t'){\dot{x}}(t')dt'+\eta (t) \end{aligned}$$

where *x*(*t*) is the position of the particle at time *t*, $\Gamma (t-t')$ is the friction kernel and $\eta (t)$ is the stochastic noise which represents the thermal agitations of the fluid molecules. Within the Jeffrey’s fluid model (generalized Maxwell model with single relaxation time), the memory kernel takes the form^9,24,32,36^

2 $$\begin{aligned} \Gamma (t-t')=2\gamma _{0}\delta (t-t')+\frac{\gamma _{1}}{\tau _{1}}\exp \left( -\frac{t-t'}{\tau _{1}}\right) \end{aligned}$$

and to satisfy the *fluctuation-dissipation theorem* (FDT) (and hence causality), the noise correlation function $\left<\eta (t)\eta (t')\right>=k_{B}T\Gamma (t-t')$; $k_{B}$ is the Boltzmann constant and *T* is the absolute temperature^32^. The first term in the expression of Eq. (2) is due to the purely viscous solvent and the second term corresponds to the polymer network present in the fluid. The noise $\eta (t)$ can be represented as $\eta (t)=\eta _{0}(t)+\eta _{1}(t)$, where $\eta _{0}(t)$ and $\eta _{1}(t)$ are two independent Gaussian random processes with mean zero and correlations $\left<\eta _{0}(t)\eta _{0}(t')\right>=2k_{B}T\gamma _{0}\delta (t-t')$ and $\left<\eta _{1}(t)\eta _{1}(t')\right>=k_{B}T\frac{\gamma _{1}}{\tau _{1}}\exp \left( -\frac{|t-t'|}{\tau _{1}}\right)$, respectively. Note that the dynamics of the particle at time *t* depends on its history and thus the process is non-Markovian. At time scales, where inertia is negligible (overdamped limit), Eq. (1) can be written as^32,37^

3 $$\begin{aligned} \gamma _{0}{\dot{x}}(t)=-\left( k+\frac{\gamma _{1}}{\tau _{1}}\right) {x(t)}+\frac{\gamma _{1}}{\tau _{1}^{2}}\int _{-\infty }^{t}\exp \left( -\frac{t-t'}{\tau _{1}}\right) x(t')dt' +\eta _{0}(t)+\eta _{1}(t). \end{aligned}$$

In order to model the system as Markovian, we introduce an auxiliary variable

4 $$\begin{aligned} X(t)=\frac{1}{\tau _{1}}\int _{-\infty }^{t}\exp \left( -\frac{t-t'}{\tau _{1}}\right) \Biggl [x(t')+ \tau _{1}\sqrt{\frac{2k_{B}T}{\gamma _{1}}}\phi _{1}(t') \Biggr ]dt', \end{aligned}$$

we can write

5 $$\begin{aligned} \begin{bmatrix}{\dot{x}}\\ {\dot{X}}\end{bmatrix}=-\begin{bmatrix}\frac{k}{\gamma _{0}}+\frac{\gamma _{1}}{\gamma _{0}\tau _{1}} &{}&{} -\frac{\gamma _{1}}{\gamma _{0}\tau _{1}}\\ -\frac{1}{\tau _{1}} &{}&{} \frac{1}{\tau _{1}}\end{bmatrix}\begin{bmatrix}x\\ X\end{bmatrix}+\begin{bmatrix}\sqrt{\frac{2k_{B}T}{\gamma _{0}}}\phi _{0} \\ \sqrt{\frac{2k_{B}T}{\gamma _{1}}}\phi _{1}\end{bmatrix} \end{aligned}$$

where $\phi _{0}$ and $\phi _{1}$ are two independent delta-correlated Gaussian random variables with mean zero and standard deviation one. If we consider,

$$\begin{aligned} {\varvec{Y}}= & {} \begin{bmatrix}x\\ X\end{bmatrix} \\ \varvec{\lambda }= & {} \begin{bmatrix}\frac{k}{\gamma _{0}}+\frac{\gamma _{1}}{\gamma _{0}\tau _{1}} &{}&{} -\frac{\gamma _{1}}{\gamma _{0}\tau _{1}}\\ -\frac{1}{\tau _{1}} &{}&{} \frac{1}{\tau _{1}}\end{bmatrix} \end{aligned}$$

and

$$\begin{aligned} {\varvec{D}}=\begin{bmatrix}D_{0} &{}&{} 0\\ 0 &{}&{} D_{1}\end{bmatrix} \end{aligned}$$

$D_{0}=\sqrt{\frac{2k_{B}T}{\gamma _{0}}}$, $D_{1}=\sqrt{\frac{2k_{B}T}{\gamma _{1}}}$, then Eq. (5) becomes a multivariate Ornstein-Uhlenbeck process^33^ and the transition probability density of the state $${\varvec{Y}}=\begin{bmatrix}x\\ X\end{bmatrix}$$, i.e., the probability density $P_{1|1}(\varvec{Y'},t'|{\varvec{Y}},t)$ of a transition from ${\varvec{Y}}$ at time *t* to $\varvec{Y'}$ at time $t'$, follows a Fokker–Planck equation (FP) $\partial _{t}P_{1|1}={\mathscr {L}}P_{1|1}$, with the Fokker–Planck operator

6 $$\begin{aligned} {\mathscr {L}}=\frac{\partial }{\partial Y_{i}}\lambda _{ij}Y_{j}+\frac{1}{2}\frac{\partial ^{2}}{\partial Y_{i}\partial Y_{j}}({\varvec{D}}{\varvec{D}}^{T})_{ij}. \end{aligned}$$

The solution of the FP is a multivariate normal distribution with mean $\varvec{\mu }(\Delta t)=\exp {(\varvec{-\lambda }\Delta t)}{\varvec{Y}}$ and covariance $\varvec{\Sigma }(\Delta t)=\varvec{\sigma } -[\exp {(\varvec{-\lambda }\Delta t)}\varvec{\sigma }\exp {(\varvec{-\lambda } ^{T}\Delta t)}]$; $\Delta t=|t'-t|$. In the limit of $\Delta t \rightarrow \infty$ one obtains the stationary probability distribution $P_{1}({\varvec{Y}})$ which clearly has a zero mean and covariance $\varvec{\sigma }=\left<{\varvec{Y}}{\varvec{Y}}^{T}\right>$. $\varvec{\lambda }$, $\varvec{\sigma }$ and ${\varvec{D}}$ are related by the stationary condition ${\mathscr {L}}P_{1}({\varvec{Y}})={\varvec{0}}$, which results into $\varvec{\lambda }\varvec{\sigma }+(\varvec{\lambda }\varvec{\sigma })^{T}={\varvec{D}}{\varvec{D}}^{T}$^33^. A straight forward calculation yields^32^

7 $$\begin{aligned} \varvec{\sigma }=\frac{k_{B}T}{k}\begin{bmatrix}1 &{}&{} 1\\ 1&{}&{} 1+\frac{k\tau _{1}}{\gamma _{1}}\end{bmatrix} \end{aligned}$$

The auto-regressive (AR) representation of the process, thus, can be written as^34^

8 $$\begin{aligned} {\varvec{Y}}_{n}=\exp {(-\varvec{\lambda }\Delta t)}{\varvec{Y}}_{n-1}+\varvec{\epsilon }_{n} \end{aligned}$$

where $\varvec{\epsilon }_{n}$ is a zero mean Gaussian random vector with co-variance $\varvec{\Sigma }(\Delta t)$.

## Bayesian inference I

If we consider the observations of the time series with *N* sample points ${\varvec{Y}}\equiv (\varvec{Y_{0}},\varvec{Y_{1}},\varvec{Y_{2}},...,\varvec{Y_{N}})$ being independently selected from the stationary distribution $P_{1}({\varvec{Y}})$, then according to the Bayes’ theorem if $\varvec{\Theta }$ is a set of unknown parameters, the conditional probability of the parameters given the observations, $P_{1}(\varvec{\Theta |Y})$ (posterior probability) is proportional to the product of the conditional probability of the observations given the parameters, $P_{1}(\varvec{Y|\varvec{\Theta }})$ (likelihood function) and the prior probability of the parameters $P(\varvec{\Theta })$^16,33,38^, i.e.,

9 $$\begin{aligned} P_{1}(\varvec{\Theta |Y}) \propto P_{1}(\varvec{Y|\Theta }) P(\Theta ) \end{aligned}$$

Therefore, for a non-informative prior probability, $\ln P_{1}(\varvec{\Theta |Y}) \propto \ln P_{1}(\varvec{Y|\Theta })$. Straightforwardly using the Gaussian nature of the stationary probability described above^16,33^,

10 $$\begin{aligned} \ln P_{1}(\varvec{\Theta |Y})=\frac{N}{2}\ln \frac{1}{4\pi ^{2}|\varvec{\sigma }|} - \frac{1}{2}\sum _{n=1}^{N}{\varvec{Y}}_{n}^{T}\varvec{\sigma }^{-1}{\varvec{Y}}_{n}. \end{aligned}$$

The maximum *a posteriori* probability (MAP) estimation is obtained by optimizing $\ln P_{1}(\varvec{\Theta |Y})$ with respect to $\varvec{\Theta }$. The corresponding standard error is obtained from the square root of the inverse of the Hessian matrix, ${\varvec{H}}=-\varvec{\nabla }\varvec{\nabla }\ln P_{1}(\varvec{\Theta |Y})$. Here, if the parameter $\Theta$ is $\varvec{\sigma }$ then the MAP estimation is

11 $$\begin{aligned} \varvec{\sigma }^{*}=\frac{1}{N}\sum _{n=1}^{N}{\varvec{Y}}_{n}{\varvec{Y}}_{n}^{T} \end{aligned}$$

Clearly, from Eqns. (7) and (11) we conclude

12 $$\begin{aligned} k^{*}=\frac{Nk_{B}T}{\sum _{n=1}^{N}x_{n}^{2}} \end{aligned}$$

with the corresponding standard error $\frac{k^{*}}{\sqrt{N}}$.

However, this method can not provide all rheological parameters involved in the process.

## Bayesian inference II

Because of the Markovian property of our process, the conditional probability of the whole sample path given a set of unknown parameters $\varvec{\Theta }$ is^16,33,38^

$$\begin{aligned} P({\varvec{Y}}|\varvec{\Theta })=\prod _{n=1}^{N-1}P_{1|1}({\varvec{Y}}_{n+1}|{\varvec{Y}}_{n},\varvec{\Theta })P_{1}({\varvec{Y}}_{1},\varvec{\Theta }) \end{aligned}$$

and similar to Bayesian inference I, the log posterior probability ($\ln P(\varvec{\Theta }|{\varvec{Y}})$) for non-informative prior information is proportional to the log likelihood function ($\ln P({\varvec{Y}}|\varvec{\Theta })$). Nevertheless, the MAP estimation by the optimization of $\ln P(\varvec{\Theta }|{\varvec{Y}})$ depend both on the particle’s position *x* and the auxiliary variable *X*^33^. Therefore, we need an appropriate likelihood function which can be calculated only using the partial observation of the process, i.e., the trajectory of the particle ${\varvec{x}}\equiv (x_{0}, x_{1},...,x_{N})$.

### Likelihood function

We can use the Kalman filter method^34,39,40^ to calculate the likelihood function from the partial observation of the process ${\varvec{Y}}$. For the *n*-th step, one can write

13 $$\begin{aligned} x_{n}=C{\varvec{Y}}_{n} \end{aligned}$$

where $$C=\begin{bmatrix}c&\,&0\end{bmatrix}$$; *c* is the calibration factor related to the experimental time-series which can be set to unity if the data is already in desired units. Using the AR structure (Eq. 8) and the partial observation of the process, the Kalman filter algorithm iteratively approaches to the likelihood $P(x_{1},...,x_{N}|x_{0},\varvec{\Theta })$ for a certain set of the unknown parameters $\varvec{\Theta }$. The log-likelihood function has the form^34^

14 $$\begin{aligned} -\ln P(x_{1},...,x_{N}|x_{0},\Theta )=\frac{1}{2}\sum _{n=1}^{N}\Biggl (\log 2\pi + \log \Omega _{n|n-1}+ \frac{(x_{n}-{\hat{x}}_{n|n-1} )^{2}}{\Omega _{n|n-1}}\Biggr ). \end{aligned}$$

where ${\hat{x}}_{n|n-1}$ and $\Omega _{n|n-1}$ are the conditional mean and variance of $x_{n}$ given $x_{1}, x_{2},...,x_{n-1}$ and $\Theta$. Further, as discussed above, the optimization of the log-likelihood function with respect to the parameters (we are interested in the set of parameters $\varvec{\Theta }=(k, \eta _{0}, \eta _{1}, \tau _{1})$) can yield the MAP estimations for the parameters. The corresponding standard error can be calculated using the Hessian matrix. For convenience, we use the Bayesian I to infer the MAP estimation of the trap stiffness, i.e., $k^{*}$ (Eq. 12) and further optimize the log-likelihood function (Eq. 14) for $\varvec{\Theta }=(\eta _{0}, \eta _{1}, \tau _{1})$. Because of a large amount of literature discussing the Kalman filter^34,39,40^, we describe it briefly in our context.

### Kalman filter

The Kalman filter algorithm for a linear system generally takes an observation model which linearly depends on the state of the system. Here, the state variable follows the linear transition equation

15 $$\begin{aligned} {\varvec{Y}}_{n}={\varvec{F}}{\varvec{Y}}_{n-1}+\varvec{\epsilon }_{n} \end{aligned}$$

where ${\varvec{F}}=\exp {(-\varvec{\lambda }\Delta t)}$ and $\varvec{\epsilon }_{n}\sim {\mathscr {N}}({\varvec{0}},\varvec{\Sigma }(\Delta t))$; the observable $x_{n}$ depends linearly on the state variable as in Eq. (13). Further, it brilliantly utilizes the Bayes’ theorem to recursively approach the likelihood function $P(x_{1},...,x_{N}|x_{0},\varvec{\Theta })$. According to Bayes’ theorem, the probability of ${\varvec{Y}}_{n}$ given the observation $x_{n}$, the previous state ${\varvec{Y}}_{n-1}$ and the parameter set $\varvec{\Theta }$ is

16 $$\begin{aligned} P({\varvec{Y}}_{n}|x_{n},{\varvec{Y}}_{n-1},\Theta )\propto P(x_{n}|{\varvec{Y}}_{n},{\varvec{Y}}_{n-1},\Theta )P({\varvec{Y}}_{n}|{\varvec{Y}}_{n-1},\Theta ) \end{aligned}$$

where $P(x_{n}|{\varvec{Y}}_{n},{\varvec{Y}}_{n-1},\varvec{\Theta })$ (*n*-th iteration for the likelihood function) is the probability of the observation $x_{n}$ given ${\varvec{Y}}_{n}$ , ${\varvec{Y}}_{n-1}$ and $\varvec{\Theta }$; and $P({\varvec{Y}}_{n}|{\varvec{Y}}_{n-1},\Theta )$ (*n*-th iteration of the prior) is the transition probability of the state ${\varvec{Y}}_{n-1}$ to ${\varvec{Y}}_{n}$ in the sampling time step $\Delta t$, given the parameter set $\varvec{\Theta }$, which is a normal distribution as discussed in the previous section. Optimization of $P({\varvec{Y}}_{n}|x_{n},{\varvec{Y}}_{n-1},\varvec{\Theta })$ should infer the expected *n*-th step of the state given the observation $x_{n}$ and parameters $\varvec{\Theta }$, i.e.,

$$\begin{aligned} \hat{{\varvec{Y}}}_{n|n}=\hat{{\varvec{Y}}}_{n|n-1}+\varvec{\omega }_{n|n-1}C^{T}(C\varvec{\omega }_{n|n-1}C^{T})^{-1}(x_{n}-C\hat{{\varvec{Y}}}_{n|n-1}) \end{aligned}$$

and the corresponding covariance, i.e.,

$$\begin{aligned} \varvec{\omega }_{n|n}=\varvec{\omega }_{n|n-1}-\varvec{\omega }_{n|n-1}C^{T}(C\varvec{\omega }_{n|n-1}C^{T})^{-1}C\varvec{\omega }_{n|n-1} \end{aligned}$$

Straightforwardly, we can update the prior probability distribution function to another normal distribution of mean $\hat{{\varvec{Y}}}_{n+1|n}={\varvec{F}}\hat{{\varvec{Y}}}_{n|n}$ and covariance $\varvec{\omega }_{n+1|n}={\varvec{F}}\varvec{\omega }_{n|n}{\varvec{F}}^{T}+\varvec{\Sigma }$. The updated likelihood probability distribution function, i.e., the probability of $x_{n+1}$ given the previous observations ($x_{n}$, $x_{n-1}$, $x_{n-2}$,...$x_{0}$) and $\varvec{\Theta }$, evidently, is a normal distribution of mean ${\hat{x}}_{n+1|n}=C\hat{{\varvec{Y}}}_{n+1|n}$ and variance $\Omega _{n+1|n}=C\varvec{\omega }_{n+1|n}C^{T}$.

Therefore, the implemented Kalman filter algorithm is the following:

17 $$\begin{aligned}&{{\varvec{\hat{Y}}}}_{n|n}={{\varvec{\hat{Y}}}}_{n|n-1}+\varvec{\omega }_{n|n-1}C^{T}(C\varvec{\omega }_{n|n-1}C^{T})^{-1}(x_{n}-C{{\varvec{\hat{Y}}}}_{n|n-1})\\&{{\varvec{\hat{Y}}}}_{n+1|n}={\varvec{F}}{{\varvec{\hat{Y}}}}_{n|n}\\&{\hat{x}}_{n+1|n}=C{{\varvec{\hat{Y}}}}_{n+1|n}\\&\varvec{\omega }_{n|n}=\varvec{\omega }_{n|n-1}-\varvec{\omega }_{n|n-1}C^{T}(C\varvec{\omega }_{n|n-1}C^{T})^{-1}C\varvec{\omega }_{n|n-1}\\&\varvec{\omega }_{n+1|n}={\varvec{F}}\varvec{\omega }_{n|n}{\varvec{F}}^{T}+\varvec{\Sigma }\\&\Omega _{n+1|n}=C\varvec{\omega }_{n+1|n}C^{T} \end{aligned}$$

To initiate the iterations, we choose $$\hat{{\varvec{Y}}}_{1|0}=\begin{bmatrix}0&\,&0\end{bmatrix}^{T}$$ and $\varvec{\omega }_{1|0}=\varvec{\Sigma }(\Delta t)$; where $\varvec{\Sigma }(\Delta t)={\varvec{D}}^{*}{\varvec{D}}^{*T}\Delta t$. Through the above described iterations we finally obtain the probability of the observation sample path

18 $$\begin{aligned} P(x_{1},...,x_{N}|x_{0},\varvec{\Theta })=\prod _{n=2}^{N}P(x_{n}|x_{m},m<n,\varvec{\Theta },x_{0}). \end{aligned}$$

Therefore, to implement this method one should use the iterations Eqns. (17) to calculate the log-likelihood function Eq. (14) and optimize with respect to the desired set of parameters.

## Numerical analysis and comparison

**Figure 1:**
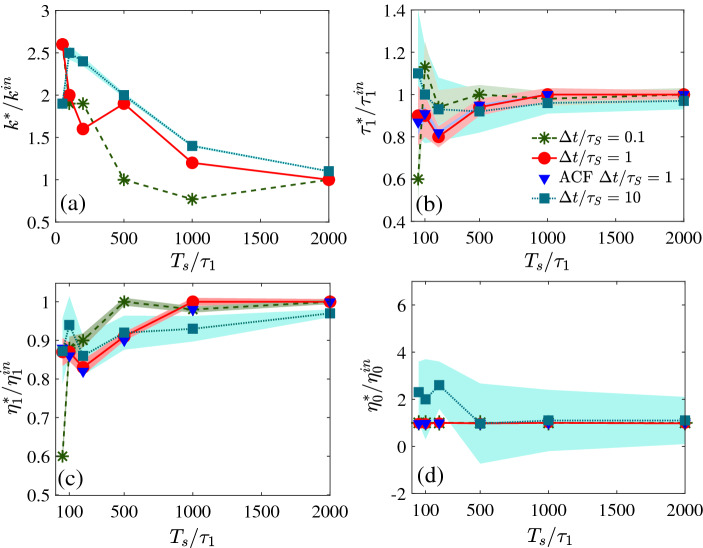
MAP estimations of the parameters using the Bayesian inference from simulated time series of various sampling time ($T_{s}$) and sampling time step ($\Delta t$) normalized by the longest ($\tau _{1}$) and the shortest ($\tau _{S}$) time scales of the process. (**a**) Estimated trap stiffness normalized by the input in the simulation ($k^{in}=0.1\,\mu$N/m) using Bayesian I. The inferences of (**b**) the time constants normalized by the input $\tau _{1}^{in}=1$ s, (**c**) the polymer viscosity divided by the corresponding input $\eta ^{in}_{1} = 0.1$ Pa s, (**d**) the solvent viscosity normalized by $\eta ^{in}_{0} = 0.001$ Pa s, using Bayesan II and the values of the trap stiffness calculated from Bayesian I. The shaded regions correspond to one standard deviations of the estimations. Clearly, when the $\Delta t$ is greater than $\tau _{S}$, the standard errors of estimations decrease with the increase of $T_{s}$. Blue triangles are the calculated parameters from the best fit of the ACF.

We numerically solve Eq. (5) with input parameters $\tau _{1}^{in}=1$ s, $\eta _{0}^{in}=0.001$ Pa s, $\eta _{1}^{in}=0.1$ Pa s, $k^{in}=0.1\times 10^{-6}$ N/m and analyze the simulated data to study the accuracy of the method in comparison to the fitting methods. Note that the smallest time scale of the process $\tau _{S}=1/\left( \frac{k}{\gamma _{0}} + \frac{\gamma _{1}}{\gamma _{0}\tau _{1}}\right)$ and the largest is $\tau _{1}$. The input parameters are chosen so that they are experimentally feasible and $\tau _{S}$ becomes 0.01 s for convenience. In Fig. 1a–d, we show the estimations of the parameters for several sampling time steps (normalized by $\tau _{S}$) with varying total sampling time ($T_{s}$) normalized by $\tau _{1}^{in}$. We estimate the trap stiffness using the Bayesian I method and use that in Bayesian II to evaluate other parameters. Note that for $\Delta t/\tau _{S}\le 1$, the MAP estimations of the parameters reach their true values with $\sim 1\%$ standard deviation when the normalized sampling time $T_{s}/\tau _{1}$ crosses $\sim 500$. Further increase in $T_{s}/\tau _{1}$, indeed, improves the precision of the result. However, if $\Delta t/\tau _{S}>1$, i.e, if the sampling time step is lower than the smallest time scale of the process, $T_{s}/\tau _{1}$ should be greater than $\sim 2000$ for reliable estimations.

### Auto-correlation function

The position auto-correlation function (ACF) of a Brownian harmonic oscillator in a viscoelastic Jeffrey’s fluid, as described in the theory section, is given by^24,32^

19 $$\begin{aligned} f(t)=\frac{1}{\nu }\Bigg [\frac{a-\frac{b}{4}(c-\nu )^{2}}{(\frac{c-\nu }{2})^{2} + (\frac{c-\nu }{2})c + \omega _{0}}\exp {\left( -\left( \frac{c-\nu }{2}\right) t\right) }+ \frac{\frac{b}{4}(c+\nu )^{2} -a}{(\frac{c+\nu }{2})^{2} + (\frac{c+\nu }{2})c + \omega _{0}}\exp {\left( -\left( \frac{c+\nu }{2}\right) t\right) }\Bigg ] \end{aligned}$$

where $a=\frac{2k_{B}T}{\gamma _{0}\tau _{1}^{2}}(1+\frac{\eta _{1}}{\eta _{0}})$, $b=\frac{2k_{B}T}{\gamma _{0}}$, $\omega _{0}=\frac{k}{\tau _{1}\gamma _{0}}$, $c=\frac{k}{\gamma _{0}}+\frac{1}{\tau _{1}}(1+\frac{\eta _{1}}{\eta _{0}})$, $\nu =\sqrt{c^{2}-4\omega _{0}}$. Ideally, the measured ACF from the position time series converges to the functional form (Eq. 19) in the limit $T_{s}\rightarrow \infty$. However, in practice, we can assume that the calculated ACF for each time lag ($t_{i}$) is Gaussian distributed around the trend $f(t_{i})$ (Eq. 19) with variance *S*, and all the measured ACF data for different time lags are independent of each other. Thus, the corresponding likelihood function is

20 $$\begin{aligned} L_{acf}=\prod _{i=1}^{N_{acf}}{\mathscr {N}}(f_{i}(\varvec{\Theta }),S). \end{aligned}$$

where ${\mathscr {N}}(f_{i}(\varvec{\Theta }),S)$ represents a normal distribution with mean $f_{i}$ and variance *S* for the i-th time lag, i.e., $t_{i}$; $N_{acf}$ is the total number of ACF data points. Optimization of the logarithm of $L_{acf}$ with respect to $\varvec{\Theta }$ provides the estimation of $\varvec{\Theta }$ with the corresponding standard deviation. Note that the least-squares fitting method also works on the same assumption and approaches to same results^38^. However, we observe that such fitting predominantly depends on the range of the data considered. In Tab. 1, we show the estimated parameters with one standard deviation by fitting the ACF measured from a simulated data of total sampling time $T_{s}/\tau _{1}=2000$ and sampling time step $\Delta t/\tau _{S}=0.1$ with the same parameters as described above as input parameters. We vary the fitting range from time lag $0-0.1$ upto $0-30$ and show that the estimated results are closest to the input parameters for fitting rang 0–0.5 to 0–1. Note that in this range the standard errors corresponding to all the parameters are also the smallest.

**Table 1:** Parameter estimated from simulated time series of $T_{s}/\tau _{1}=2000$ and time step $\Delta t/\tau _{S}=0.1$ by fitting the corresponding ACF over various range of time lags. Clearly, the estimations are close to all the input parameters when the fitting range lies in between from 0–0.5 to 0–1 s. The input parameters in the simulation are the same as in Fig. 1.

Fitting range (s)	$\eta _{0}^{*}\pm 1\sigma$ (mPa s)	$\eta _{1}^{*} \pm 1\sigma$ (mPa s)	$\tau _{1}^{*}\pm 1\sigma$ (s)
0–0.1	$1.0\pm 0.0$	$440.0\pm 0.1$	$4.6\pm 2.0\times 10^{-3}$
0–0.5	$1.0\pm 1.0\times 10^{-3}$	$100\pm 130\times 10^{-3}$	$1.0\pm 0.8\times 10^{-3}$
0–1	$1.1\pm 2.4\times 10^{-3}$	$110\pm 15.0\times 10^{-3}$	$1.1\pm 0.8\times 10^{-3}$
0–5	$5.0\pm 0.1$	$110.0\pm 0.1$	$1.1\pm 0.2\times 10^{-3}$
0–10	$2.5\pm 0.0$	$100.0\pm 0.0$	$1.1\pm 1.0\times 10^{-3}$
0–20	$30.0\pm 0.1$	$70.0\pm 0.1$	$1.3\pm 5.0\times 10^{-3}$
0–30	$50.0\pm 0.1$	$60.0\pm 0.1$	$1.9\pm 13.0\times 10^{-3}$

**Figure 2:**
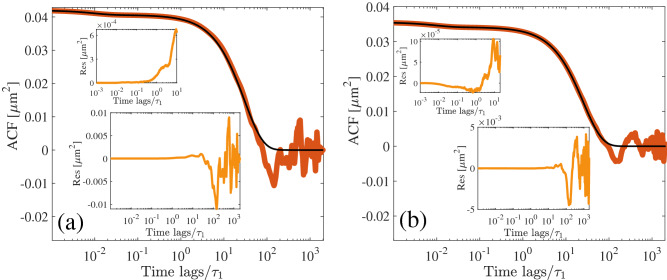
ACF for simulated time series of time step $\Delta t/\tau _{S}=0.1$ and (**a**) $T_{s}/\tau _{1}=2000$, (**b**) $T_{s}/\tau _{1}=10000$ with the corresponding best fit and residue in the insets. Upper inset in each figure concentrates on the initial regions of the residuals which shows that the variance *S* of the model increases after a certain time lag.

To better understand the discrepancies in the obtained parameter values for different fitting ranges, we plot the ACF with the corresponding best fit (where the standard deviations are least) and residue in Fig. 2a (from simulated data of $T_{s}/\tau _{1}=2000$ and $\Delta t/\tau _{S}=0.1$) and Fig. 2b (from simulated data of $T_{s}/\tau _{1}=10000$ and $\Delta t/\tau _{S}=0.1$). Vividly, the central assumptions of the method, i.e., Gaussian distribution around the trend and the constant variance of the residue (*S*) are not valid over the whole range of time lag. After a certain time lag the variance of the residue increases exceedingly. The best fitting range increases with the increase in the total sampling time. Therefore, to make this process consistent, further processing of the data, such as careful binning is required^41^. Note that binning may lose information hidden in short times. If the sampling time step is much smaller compared to the smallest time scale $\tau _{S}$ of the system then the binning can improve the fitting significantly. The parameter values obtained from the best fitting of the ACF are very close to those calculated using the Bayesian method developed in this paper (as shown in Fig. 1b–d).

## Experiment

**Figure 3:**
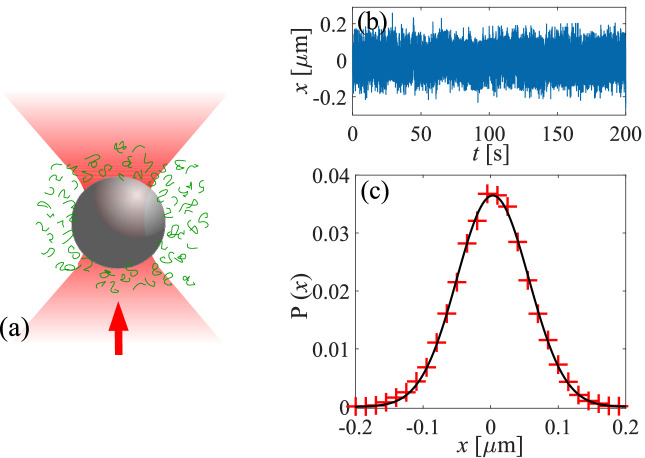
(**a**) Schematic diagram of a trapped spherical probe using optical tweezers in a viscoelastic medium. Arrow shows the direction of the trapping laser beam. (**b**) Representative experimental trajectory of $T_{s}=200$ s and $\Delta t=0.002$ s of a confined particle (radius 1.95 μm) in an optical trap of trap stiffness $k=1.4\pm 0.1~\upmu~\text{N}/\text{m}$ in $0.02~\%\text{w}/\text{w}$ PAAM to water mixture and, (**c**) the corresponding probability distribution.

As viscoelastic fluids, we use an aqueous solution of polyacrylamide (Polysciences, PAAM, $M_{w}=18\times 10^6$ gm $mol^{-1}$) at different concentrations. Note that the concentrations of the PAAM strongly dictates the viscoelastic parameters of the solution^25,42^. A very small volume fraction ($\sim 0.01$) of spherical polystyrene particles (radius $1.95 \mu$m) are added to the PAAM-water solution as probes. For sample making, we seal the suspension between two glass slides with a spacing of 100 $\mu$m. We kept this sample cell in contact with a thermal bath kept at $25^\circ$C. We trap one of the spherical probe particles in the sample fluid far away from any glass surface (to avoid wall effects) using an optical tweezers built by tightly focused a Gaussian infra red (IR, $\lambda =1064$ nm) laser beam with a high numerical aperture ($N.A.=1.3$) oil immersion microscope objective. We use a CMOS camera to record videos for the thermal position fluctuation of the probe particle at a frequency of 500 Hz. The position of the particle are tracked from the videos using MATLAB by standard particle tracking algorithm. For optimization of the log-likelihood function, we use an inbuilt MATLAB optimization tool. In Fig. 3a, we show a schematic of the setup, and Fig. 3b–c represent a typical position time series of the probe particle and the corresponding probability distribution respectively.

## Data and analysis

**Figure 4:**
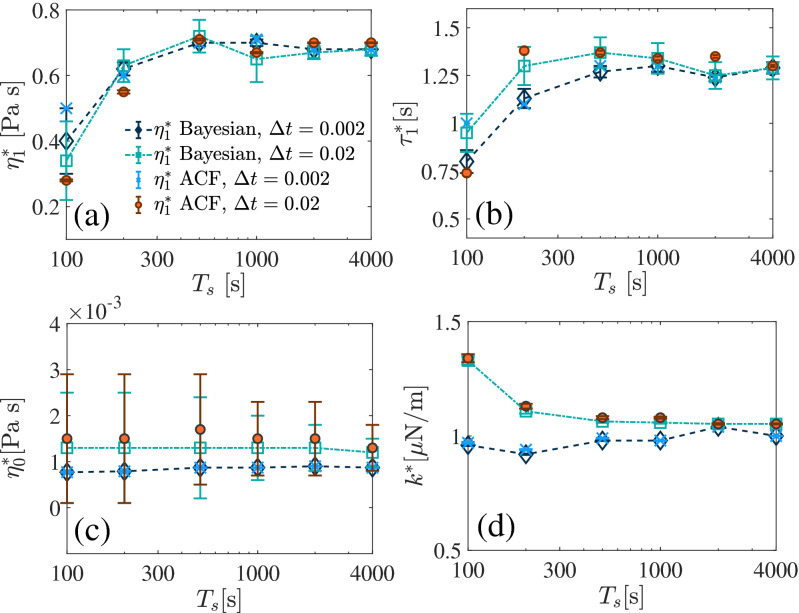
MAP estimations of the parameters from the thermal position fluctuations of a trapped particle in a viscoelastic fluid ($0.06~\% \text{w}/\text{w}$ PAAM-water) for various $T_{s}$ and $\Delta t$.

For all measurements in this work, we maintain the trapping laser power fixed which results in trap stiffness $k=(1-1.6)~\mu$N/m. Note that, the PAAM concentration may slightly change the refractive index of the fluid, which can bring a small modification to the trap stiffness. The trap stiffness is kept relatively lower to avoid any potential coupling of the trap with the fluid^43^. To experimentally check the dependency of the Bayesian method on the data length and the sampling time step, we analyze data for sampling times ranging from 100 to 4000 s and sampling time steps $\Delta t=0.002$ s and 0.02 s. We show representative data ($0.06~\%$ w/w PAAM-water) in Fig. 4a–d for the estimations of $\eta _{1}\,\tau _{1},\,\eta _{0}$ and *k* respectively. Clearly, after $T_{s}=200$ s, the estimations are saturating for both the values of $\Delta t$. However, the precision and accuracy of the measurements are higher for $\Delta t=0.002$ s. The standard error of the estimations, as expected, decreases with increasing $T_{s}$. Interestingly, the best fittings of the auto-correlation functions evaluate approximately the same values as obtained from the Bayesian method, and exhibits similar behavior.

**Figure 5:**
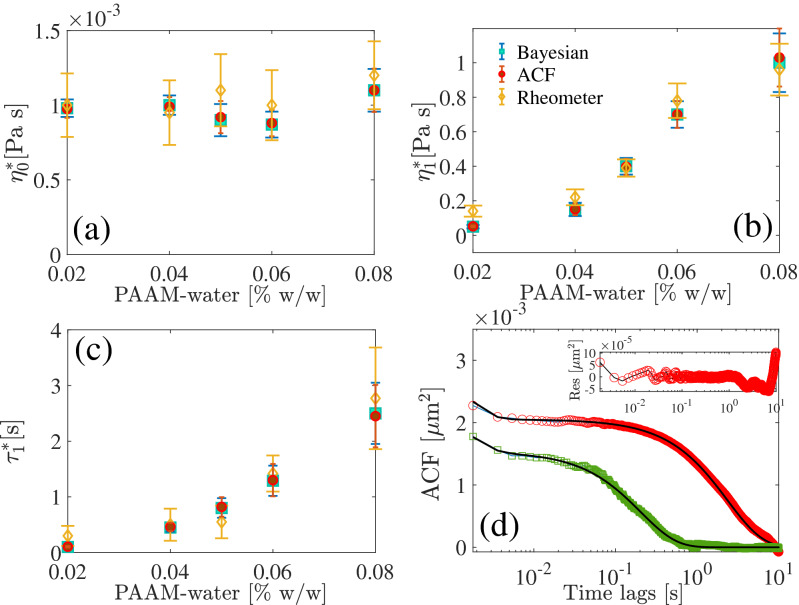
(**a**)–(**c**) Estimated parameters for various concentration of water PAAM solution. (**d**) ACFs for PAAM-water concentrations 0.02% w/w (green squares) and 0.06% w/w (red circles). (inset) Residue of the fitting for 0.06% w/w PAAM-water concentration. The black lines represent the best fittings.

As expected, the experimental data also shows the same strong dependence of the ACF fitting method on the range of data used for the fitting, as observed for numerically obtained ACFs. This verifies our theoretical understandings about the fitting method as described in the theory section. In Fig. 5d, we show the residue (inset) of the best fitting which clarifies that the assumption of the Gaussian distribution around the trend and the constant variance is only valid over the initial range of time lags.

In order to verify the estimated results obtained from the Bayesian method, we perform the experiments with various (0.02–0.08% w/w) PAAM-water solutions and compare with the parameters obtained from the bulk rheology using a commercial rheometer (DHR-2-TA Instruments). We fit the frequency dependent viscosity measured using the rheometer with the corresponding theoretical expression for Jeffrey’s fluid^24^ to estimate the parameters. We observe (Fig. 5a–c) a very good agreement for all the three methods (Bayesian, best fitting of the ACF and rheometer measurements). The $\eta _{0}$ remains close to the viscosity of water at $25^{\circ }$C and approximately independent of the concentrations, however, $\tau _{1}$ and $\eta _{1}$ increases with the increase of the PAAM concentration. The similar trend is also observed previously in several works^25,42^. The standard errors are calculated over several independent measurements.

## Conclusion

In conclusion, we present a Bayesian inference based technique to measure the rheological parameters of a Jeffrey’s fluid. We show that the estimations from the proposed technique are more reliable compared to that obtained from the auto-correlation function. The parameter estimations from the ACF depend very sensitively on the range over which the data is fitted. We define the best fitting when the standard deviations corresponding to the estimations are minimum. At the best fitting, the estimations from the fitting method and the proposed method are approximately same. Further, to verify our method, we compare our inferred parameter values for various viscoelastic fluids prepared by mixing PAAM into water in different concentrations, with that measured using a commercial rheometer and obtain a strong agreement. We also observe that for a reliable measurement, the data should have sampling time step comparable to the shortest time scale of the system and total sampling time larger than $\sim 200$ times the time constant of the fluid. The established method is comparatively fast (as it does not need further processing of the data), advanced, more reliable, and less affected by systematic noises. It uses less inputs from the user than any other least-squares fitting methods as it efficiently utilizes the information hidden in the data. Even though the Jeffrey’s fluid model is a particular simple description of viscoelastic fluids, there exist many examples where such approach has been experimentally confirmed^8–15^. It should be mentioned, however, that the concept of Bayesian inference can be also applied to more complex models involving more than a single stress relaxation time (even though the accuracy of the method generally becomes worse progressively with the introduction of additional parameters). The standard Bayesian model comparison techniques can be used to choose an appropriate model for a given data to avoid unnecessary generalization. Also, it can be extended for free Brownian probes and active Brownian particles.
